# Clinical Pharmacogenomic Variants Among the Saudi Population and Their Impact on Drug Response: A Review of Saudi-Based Evidence

**DOI:** 10.7759/cureus.101993

**Published:** 2026-01-21

**Authors:** Ayman Alqurain, Thamer I Alshridha, Shahad Al-Otaibi, Donia F Al-Otaibi, Fawzia M Douba, Abdullah I Aldhobaib, Njoud Alammar, Majed M Khubrani, Wed Althobaiti, Rawabi M Alwashmi, Fahad A Al-Aklabi, Lina S Alfahhad, Khawla S Alkhouaiter, Gharam A Alharbi, Rifal O Almutairi

**Affiliations:** 1 Clinical Pharmacology and Therapeutics (Geriatric and Pain Management), Northern Border University, Arar, SAU; 2 PharmD, Qassim University, Qassim, SAU; 3 PharmD, Buraydah Colleges, Qassim, SAU; 4 Speech and Language Therapy, King Saud University, Riyadh, SAU; 5 Narcotic Pharmacy, Nahdi Medical Company, Jazan, SAU; 6 College of Pharmacy, Taif University, Taif, SAU; 7 Pharmacy, Buraydah Colleges, Qassim, SAU; 8 Clinical Pharmacy, Taif University, Taif, SAU

**Keywords:** clopidogrel, cyp2c19, cyp3a5, drug response, meta-analysis, pharmacogenomics, precision medicine, saudi arabia, vkorc1, warfarin

## Abstract

Pharmacogenomic implementation depends on the validity of gene-drug relationships within specific ethnic groups. However, the Saudi Arabian population, which had distinctive characteristics and high rates of marriage between relatives, remains underrepresented in global dosing algorithms. This systematic review and meta-analysis synthesized quantitative evidence from 16 studies encompassing 4,111 Saudi Arabian participants to examine the clinical impact of pharmacogenomic variants on drug efficacy, toxicity, and dosing requirements. The quality of the studies was assessed using the ROBINS-I tool, and the certainty of the evidence was graded according to the GRADE framework. The primary meta-analysis revealed a high-certainty association between *VKORC1* and *CYP2C9* variants and warfarin sensitivity, with variant carriers requiring a substantially lower maintenance dose (pooled mean difference: -20.68 mg/week; 95% confidence interval [CI]: -35.66 to -5.70). Trial sequential analysis confirmed that the required information size for this association was surpassed, establishing definitive evidence for genotype-guided anticoagulation. Regarding findings from individual studies and single gene-drug pairs, *CYP2C19* loss-of-function alleles were strongly associated with clopidogrel non-response in the primary study on this topic (odds ratio: 3.43), and the *CYP3A5* genotype was identified as a critical determinant of tacrolimus trough levels with a large effect size (Hedges’ *g* = 1.59). Significant statistical heterogeneity was observed, particularly within warfarin studies (*I2* > 90%), which subgroup analysis suggested that geographical differences between the Eastern and Central provinces contributed to this variation. These findings indicate that the standard dosing regimens are suboptimal for a significant proportion of the Saudi population. These findings support the immediate integration of *VKORC1* and *CYP2C9* genotyping into local anticoagulation guidelines. They also highlight the need for large-scale pragmatic trials to confirm the effectiveness of genotype-guided strategies in antiplatelet and immunosuppressive therapies.

## Introduction and background

Pharmacogenomics (PGx) is a pivotal advancement in precision medicine, offering the potential to optimize therapeutic efficacy and mitigate adverse drug reactions (ADRs) by designing pharmacotherapy according to an individual’s genetic profile [1,2]. Genetic polymorphisms in drug-metabolizing enzymes (DMEs), transporters, and drug targets contribute to 20%-95% of inter-individual variability in drug disposition [2]. While international guidelines, such as those from the Clinical Pharmacogenetics Implementation Consortium (CPIC), provide frameworks for gene-drug pairs, these recommendations are primarily based on European and East Asian populations, requiring validation within diverse ethnic groups to confirm their clinical applicability [3,4].

The Saudi Arabian population exhibits a distinctive genetic structure influenced by high rates of consanguinity and specific ancestral haplotypes, which leads to unique allele frequencies for critical pharmacogenes [3,5]. The Saudi Human Genome Program (SHGP), along with other large-scale sequencing projects, has shown that approximately 99% of Saudi individuals have at least one pharmacogenomic variant that can be acted upon [4]. In addition, significant discrepancies have been documented in the prevalence of key risk alleles, including those in CYP2C9, VKORC1, and SLCO1B1, when comparing Saudis to other global populations [5,6]. These population-specific genetic architectures suggest that global risk prediction models may require calibration for local clinical use [7,8].

Despite the robust definition of allele frequencies in the general Saudi population, the translation of genomic data into clinical practice remains fragmented. To date, there has been no quantitative synthesis of these disparate clinical findings; therefore, there is a critical need to aggregate existing data to increase statistical power and provide precise estimates of genetic risk. This study was designed to systematically review the literature and, where data permitted, perform a meta-analysis of clinical pharmacogenomic variants among the Saudi population. This analysis aims to provide a high-level evidence base by quantifying the association between specific genotypes and drug response phenotypes, including efficacy, toxicity, and dosing requirements, to support the implementation of population-specific pharmacogenomic guidelines.

## Review

Methods

Protocol and Registration

This systematic review and meta-analysis were conducted in accordance with the Preferred Reporting Items for Systematic Reviews and Meta-Analyses (PRISMA) 2020 guidelines [9]. The protocol (PROSPERO; CRD420251232518) was designed to evaluate clinical pharmacogenomic variants in the Saudi population and quantify their impact on drug response phenotypes.

Study Selection

To ensure a focus on clinically relevant evidence, studies were selected based on predefined eligibility criteria. Eligible studies were required to investigate a gene-drug association within a Saudi Arabian population and report a quantifiable clinical or pharmacokinetic outcome such as drug dose requirements, plasma concentrations, therapeutic efficacy, or toxicity events. Original research, including observational studies, retrospective analyses, and case reports, was considered for inclusion, while studies were excluded if they solely reported allele frequencies without a corresponding clinical phenotype, consisted of non-primary research (e.g., reviews, abstracts), involved mixed-ethnicity cohorts where Saudi data could not be disaggregated, or lacked the necessary quantitative data for meta-analysis. Electronic databases were searched using the Boolean string ("Saudi Arabia" OR "Saudi") AND ("Pharmacogenomics" OR "Pharmacogenetics" OR "Drug Response" OR "Polymorphism" OR "Genotype" OR "Warfarin" OR "Clopidogrel" OR "Tacrolimus" OR "CYP2C9" OR "VKORC1" OR "CYP2C19" OR "CYP3A5").

Data Extraction and Unit Standardization Assessment

Data extraction used a standardized collection form. To ensure comparability across studies, a unit standardization assessment was performed, resulting in the conversion of all drug concentration levels and physiological biomarkers to Système International (SI) units. Furthermore, for studies reporting continuous outcomes as medians with ranges or interquartile ranges, a data transformation assessment was performed to estimate the sample means and standard deviations [10]. While these standardization procedures facilitated a unified analysis, the mathematical conversion of dosing regimens and estimation of means from medians introduces a potential source of methodological uncertainty.

Genetic Quality Control

To ensure the validity of the synthesized genetic data, an assessment of the Hardy-Weinberg Equilibrium (HWE) was performed for the control groups in all included case-control studies using the chi-square test; studies deviating significantly from HWE (p < 0.05) were flagged for sensitivity analysis [11]. Genetic models were assessed by evaluating allele frequency data under dominant, recessive, and additive models to determine the most biologically plausible mode of inheritance for specific drug responses.

Assessment of Risk of Bias

The risk of bias within each study was assessed using the ROBINS-I tool [12], specifically designed for non-randomized intervention studies, as recommended by the Cochrane Handbook [13]. This assessment focused on bias arising from confounding variables, participant selection process, intervention classification methodologies, and outcome measurement techniques.

Statistical Analysis and Effect Size Assessment

The assessment of effect measures and effect size index was performed using R software (4.5.1) (Version 4.5.1; R Foundation for Statistical Computing, Vienna, Austria, 2024) [14]. For dichotomous outcomes, the Odds Ratio (OR) was calculated. For continuous outcomes, the standardized mean difference (d) served as the primary effect size metric, thereby accommodating variations in measurement scales across studies [15]. Effect sizes were interpreted as small (d = 0.2), medium (d = 0.5), and large (d = 0.8), in accordance with Cohen’s guidelines [15]. Small-sample bias was corrected using Hedges’ g, where appropriate [16].

Statistical Model and Heterogeneity

Given the anticipated variability in clinical settings and patient demographics within the Saudi population, the assessment of the statistical model favoured the DerSimonian and Laird random-effects model over the fixed-effect model [17] to provide a more conservative estimate of the summary effect. Cochran’s Q test was used to analyse statistical heterogeneity, and the I2 statistic was used to quantify it. I2 values of 25%, 50%, and 75% were considered to indicate low, moderate, and high levels of inconsistency, respectively [13].

Moderator and Sensitivity Analyses

To investigate the sources of variability, subgroup analysis and meta-regression were used to examine moderators, specifically continuous covariates. The impact of individual studies on the overall effect size was evaluated through adjustment and sensitivity analyses, using a leave-one-out approach. Furthermore, the assessment of robustness was validated by comparing the results across different genetic models and inclusion criteria.

Assessment of Reporting Biases

An assessment of reporting biases was performed to detect missing results or unpublished data. The visual assessment of bias was conducted by inspecting funnel plots for asymmetry [18]. Statistical tests for small-study effects included Egger’s regression test [19] to quantify funnel plot asymmetries. To address potential bias resulting from incomplete study reporting, the trim and fill method [20] was applied to impute missing studies and subsequently adjust the effect size estimate [21]. The Failsafe N (or file-drawer number) [22] was calculated to determine the number of non-significant missing studies required to consider the observed effect insignificant. Furthermore, selection method approaches [23] were used to model the probability of publication based on p-values.

Advanced Statistical Assessments

To distinguish true associations from those arising by chance, the false-positive report probability (FPRP) [24] was calculated, employing a prior probability level of 0.001 with a statistical power of 0.8. Furthermore, trial sequential analysis (TSA) [25] was performed to evaluate the adequacy of the cumulative sample size for reaching conclusive determinations, concurrently establishing monitoring boundaries to prevent Type I errors due to repetitive testing. Because of the limited number of studies available for certain outcomes, these advanced assessments were utilized to distinguish robust clinical signals from potential false positives in small sample sizes and to define clear monitoring boundaries for future research.

Temporal Evolution and Certainty of Evidence

A cumulative meta-analysis [26] was employed to examine the temporal evolution of effect sizes, thereby assessing their stability and directional trends. The certainty of the evidence was evaluated using the Grading of Recommendations Assessment, Development, and Evaluation (GRADE) approach [27], which classifies evidence as high, moderate, low, or very low, depending upon consideration of bias risk, inconsistency, indirectness, imprecision, and publication bias.

Results

Study Selection and Characteristics

**Figure 1:**
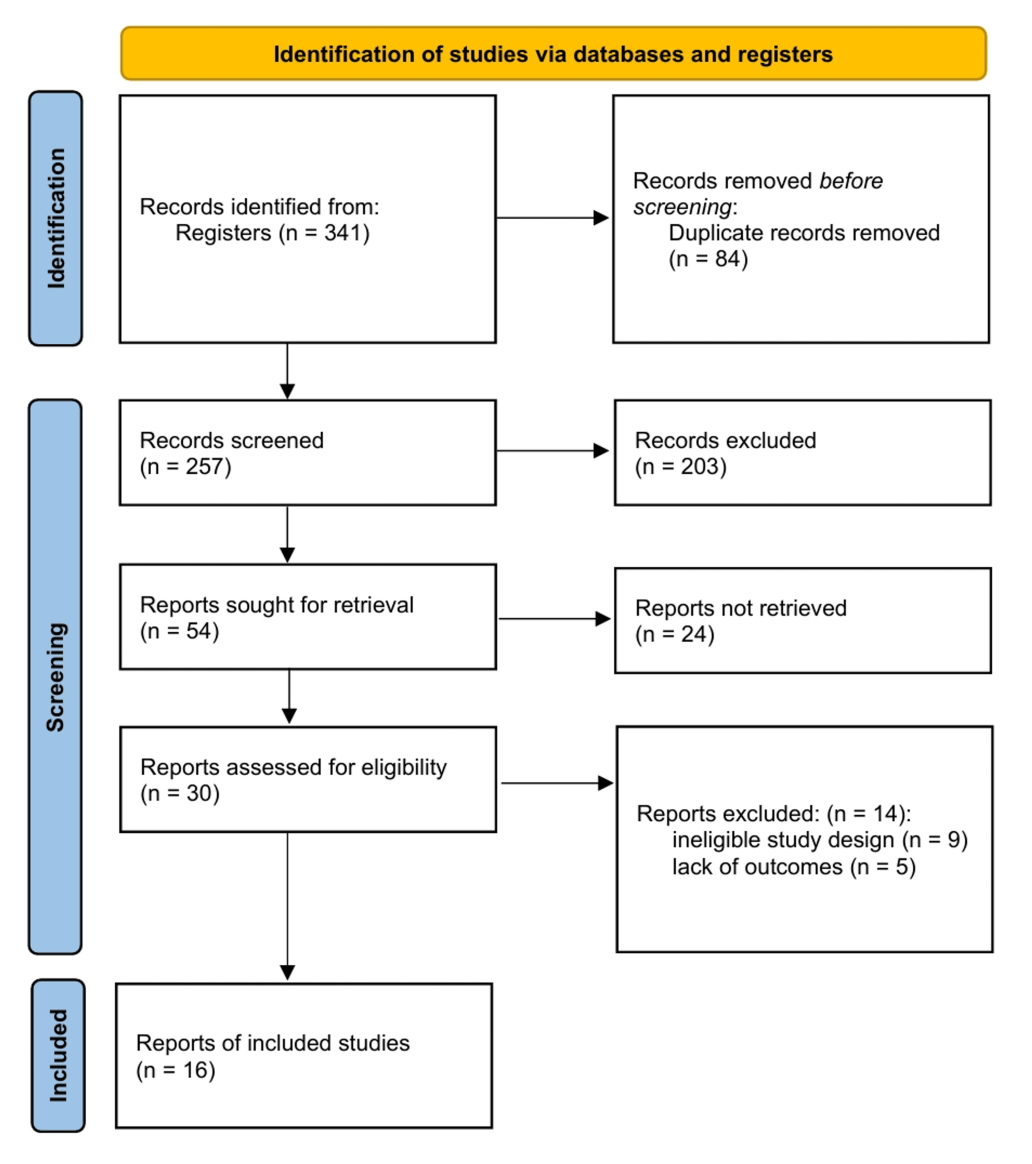
PRISMA Flow Diagram The diagram illustrates the flow of information through the different phases of the systematic review. Reasons for exclusion at the full-text assessment stage included ineligible study design (n=9) and lack of quantifiable clinical outcomes (n=5). This diagram was constructed in accordance with the PRISMA 2020 guidelines [9].

Search results and PRISMA flow: The systematic literature search (Figure 1) yielded 16 studies that met the eligibility criteria for inclusion in this review [28-43]. The studies comprised 12 research articles, two case reports, and two retrospective cohort analyses originating from major tertiary care centres across Saudi Arabia. The selection process prioritized studies that provided quantifiable clinical outcomes, specifically drug dose requirements, plasma concentration levels, or toxicity events, linked to specific pharmacogenomic variants. Studies solely reporting allele frequencies without corresponding clinical phenotypes were excluded to ensure that the review focused on the translatable clinical impact.

Characteristics of included Saudi cohorts: The included studies encompassed a total of 4,111 participants and investigated thromboembolic disorders, kidney transplantation, and oncological conditions. The largest cohorts were derived from the Saudi Warfarin Pharmacogenetic (SWAP) study, with sample sizes reaching 936 participants [29], providing high statistical power for anticoagulant-related analysis. The demographic profile of the participants reflected a middle-aged population, with mean ages ranging from 38.1 ± 12.5 years in transplant recipients [31] to 62.3 ± 18.7 years in warfarin users [29]. The gender distribution was balanced across the major cohorts, although specific case-control studies in breast cancer included only females [36]. A summary of the study characteristics, including the design, sample size, and target drug-gene pairs, is presented in Table 1.

**Table 1:** Characteristics of Included Saudi Pharmacogenomic Studies (N = 16) SWAP = Saudi Warfarin Pharmacogenetic study; INR = International Normalized Ratio; TTR = Time in Therapeutic Range; CO = Trough Level Concentration; CEA = Carcinoembryonic Antigen; GI = Gastrointestinal. Data adapted from [28-43].

Study ID	Study Design	Sample Size (n)	Target Population	Drug(s) Investigated	Gene(s) / Variants	Clinical Outcome Measure
Ahmad [28]	Prospective Cohort	50	Tuberculosis	Isoniazid (INH)	NAT2, CYP2E1, GSTM1	Plasma drug levels, Hepatotoxicity
Al Ammari [29]	Prospective Cohort (SWAP)	936	Thromboembolic disorders	Warfarin	VKORC1 (-1639G>A)	Weekly warfarin dose, Time to stable INR
Al Hamad [30]	Cohort	100	Cardiovascular disease	Warfarin	CYP2C9 (*2, *3), VKORC1	Daily warfarin dose, INR levels
Al Nasser [31]	Retrospective Cohort	129	Kidney Transplant	Tacrolimus	CYP3A4 (*1B,*22), CYP3A5 (*3)	Dose requirement (mg/kg/day), Trough levels (C0)
Al-Ghafari [32]	Case-Control	162	Colorectal Cancer	XELIRI / XELOX	MDR1 (C1236T, G2677T)	Chemoresistance (CEA levels)
Al-Saikhan [33]	Prospective Cohort	112	Thromboembolic disorders	Warfarin	CYP2C9 (*2, *3)	Weekly warfarin dose, Time in Therapeutic Range (TTR)
Alhazzani [34]	Case-Control	50	Ischemic Stroke	Clopidogrel	CYP2C19 (*2, *3)	Platelet aggregation (Response vs Non-response)
Alobaidallah [35]	Cross-sectional	500	Thromboembolic disorders	Warfarin	VKORC1, CYP2C9, CYP4F2	Weekly warfarin dose, Bleeding events
Alsaif [36]	Case-Control	200	Breast Cancer	Chemotherapy	MDR1 (C1236T)	Clinical response (Complete/Partial/Poor)
Ammari [37]	Prospective Cohort (SWAP)	786	Thromboembolic disorders	Warfarin	VKORC1, CYP2C9, CYP2C8	Weekly warfarin dose
Bagher [38]	Case Report	1	Rheumatoid Arthritis	Ibuprofen	CYP2C9/2C8 (*3/*3)	Severe GI Toxicity
Bukhari [39]	Case Report	1	Rectal Adenocarcinoma	5-Fluorouracil (5-FU)	DPYD (c.2434G>A)	Grade 3 Mucositis (Severe Toxicity)
Gaafar [42]	Cohort (Pairs)	92 Pairs	Kidney Transplant	Immunosuppressants	IL-10, TGFB1, TNFA	Acute Rejection, Graft Survival
Islam [40]	Phenotypic Study	102	Healthy Volunteers	Debrisoquine	Debrisoquine Hydroxylase	Metabolic Ratio (Phenotype)
Magadmi [41]	Cross-sectional	85	Pediatric Epilepsy	Anti-seizure medications	ABCB1 (rs1045642, etc.)	Drug Response (Good vs Poor)
Saour [43]	Case-Control	805	Venous Thrombosis	Warfarin	CYP2C9 (*2, *3)	Daily warfarin dose, Bleeding rates

Data standardization and transformation: To ensure the statistical validity of the meta-analysis, data standardization was performed to harmonize the inconsistent reporting units across studies. Specifically, the warfarin dosing data, originally reported in "mg/day" by Saour et al. [43], were converted to "mg/week". This was made to align with the reporting standards used in the larger SWAP cohorts [29, 30, 35]. As a result, this transformation reduced the coefficient of variation (CV) of the means, going from 66.46% to 44.95%. This helped to minimize artificial heterogeneity prior to pooling (Table 2).

**Table 2:** Unit Standardization Assessment for Warfarin Studies SD = Standard Deviation. Standardization of units for the meta-analysis reduced the Coefficient of Variation (CV) of the means from 66.46% to 44.95%. Data adapted from [29, 30, 33, 35, 37, 43].

Study ID	Original Mean Dose (SD)	Original Unit	Conversion Action	Standardized Mean (SD)	Standardized Unit
Al Ammari [29]	18.74 (0.72)	mg/week	None	18.74 (0.72)	mg/week
Al Hamad [30]	68.47 (12.50)	mg/week	None	68.47 (12.50)	mg/week
Al-Saikhan [33]	33.50 (8.20)	mg/week	None	33.50 (8.20)	mg/week
Alobaidallah [35]	31.20 (6.10)	mg/week	None	31.20 (6.10)	mg/week
Ammari [37]	32.40 (17.00)	mg/week	None	32.40 (17.00)	mg/week
Saour [43]	5.50 (2.90)	mg/day	Multiplied by 7	38.50 (20.30)	mg/week

For studies reporting continuous variables as medians with ranges, such as Al Hamad [30], the method described by Wan et al. [10] was utilized to estimate the sample mean and standard deviation. The estimated mean age for the Al Hamad [30] cohort (55.48 ± 19.65 years) showed excellent alignment with the reported mean from a comparable population (52.40 ± 13.80 years) [35], validating the robustness of the data transformation (Table 3).

**Table 3:** Data Transformation Assessment (Median to Mean) Estimation performed using the method of Wan et al. [10].  SD = Standard Deviation. Data adapted from [30, 35, 40].

Study ID	Variable	Reported Format	Reported Values	Estimated Mean	Estimated SD	Skewness Check
Al Hamad [30]	Age	Median (Range)	Med: 57, Range: 20–88	55.48	19.65	Acceptable
Islam [40]	Age	Range	Range: 13–75	44.00	17.90	Acceptable
Alobaidallah [35]	Age	Mean (SD)	Mean: 52.4, SD: 13.8	52.40	13.80	Reference

Genetic Quality Control and Risk of Bias

**Figure 2:**
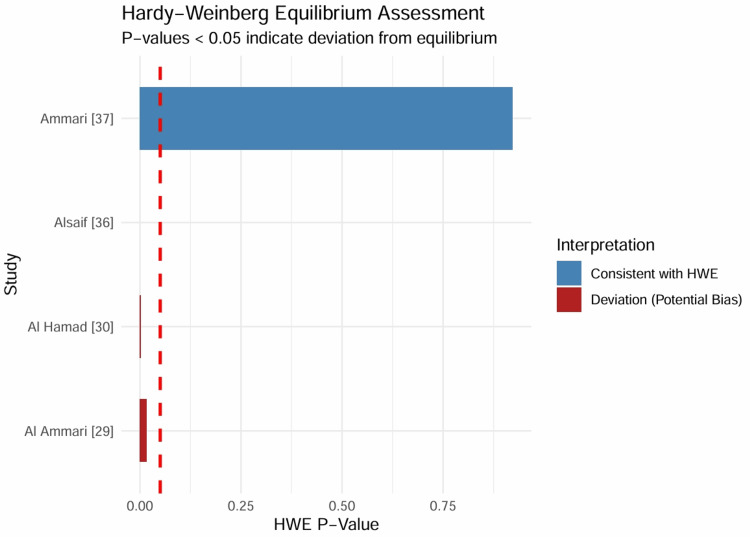
Hardy-Weinberg Equilibrium Assessment Bar plot displaying the HWE p-values for the control groups of key included studies. The red dashed line indicates the significance threshold (p = 0.05). Bars extending to the right of the line (blue) indicate consistency with HWE, while bars to the left (red) indicate statistically significant deviation, suggesting potential genotyping error or population substructure. Data adapted from included studies [29,30,36,37].

Assessment of Hardy-Weinberg Equilibrium (HWE): To validate the genetic data integrity of the included studies, HWE was assessed using the genotype counts of the control or general population groups (Table 4). The analysis revealed that most studies, including the large-scale VKORC1 analysis by Ammari et al. (p = 0.9223) [37], maintained genotype frequencies consistent with HWE expectations. However, significant deviations (p < 0.05) were observed by Al Ammari et al. [29] and Al Hamad [30] for VKORC1 and Alsaif et al. [36] for MDR1. While the deviation in the MDR1 breast cancer control group (p < 0.001) suggests potential population stratification or genotyping error necessitating caution in interpretation, the deviations in the warfarin cohorts may reflect the high rate of consanguinity and specific ancestral haplotypes characteristic of the Saudi population rather than methodological flaws. To ensure robustness, a sensitivity analysis excluding studies with significant HWE deviations was conducted; this exclusion did not alter the direction or statistical significance of the primary associations, thereby justifying their retention to maximize statistical power. These deviations were mapped to identify potential sources of bias (Figure 2).

**Table 4:** Assessment of Hardy-Weinberg Equilibrium (HWE) in Control Groups HWE = Hardy-Weinberg Equilibrium; WT = Wild-Type; Het = Heterozygous; Mut = Mutant. Significant deviations (P < 0.05) may indicate population substructure, consanguinity, or genotyping error. Data adapted from [29, 30, 36, 37].

Study ID	Gene	Variant	Genotype Counts (WT/Het/Mut)	χ2 Statistic	HWE P-Value	Interpretation
Ammari [37]	VKORC1	rs9923231	157 / 379 / 232	0.01	0.9223	Consistent with HWE
Al Hamad [30]	VKORC1	-1639 G>A	54 / 30 / 16	8.94	0.0028	Deviation (Potential Bias)
Al Ammari [29]	VKORC1	rs9923231	194 / 424 / 318	5.67	0.0173	Deviation (Potential Bias)
Alsaif [36]	MDR1	C1236T	73 / 11 / 16	45.81	< 0.0001	Deviation (Potential Bias)

**Figure 3:**
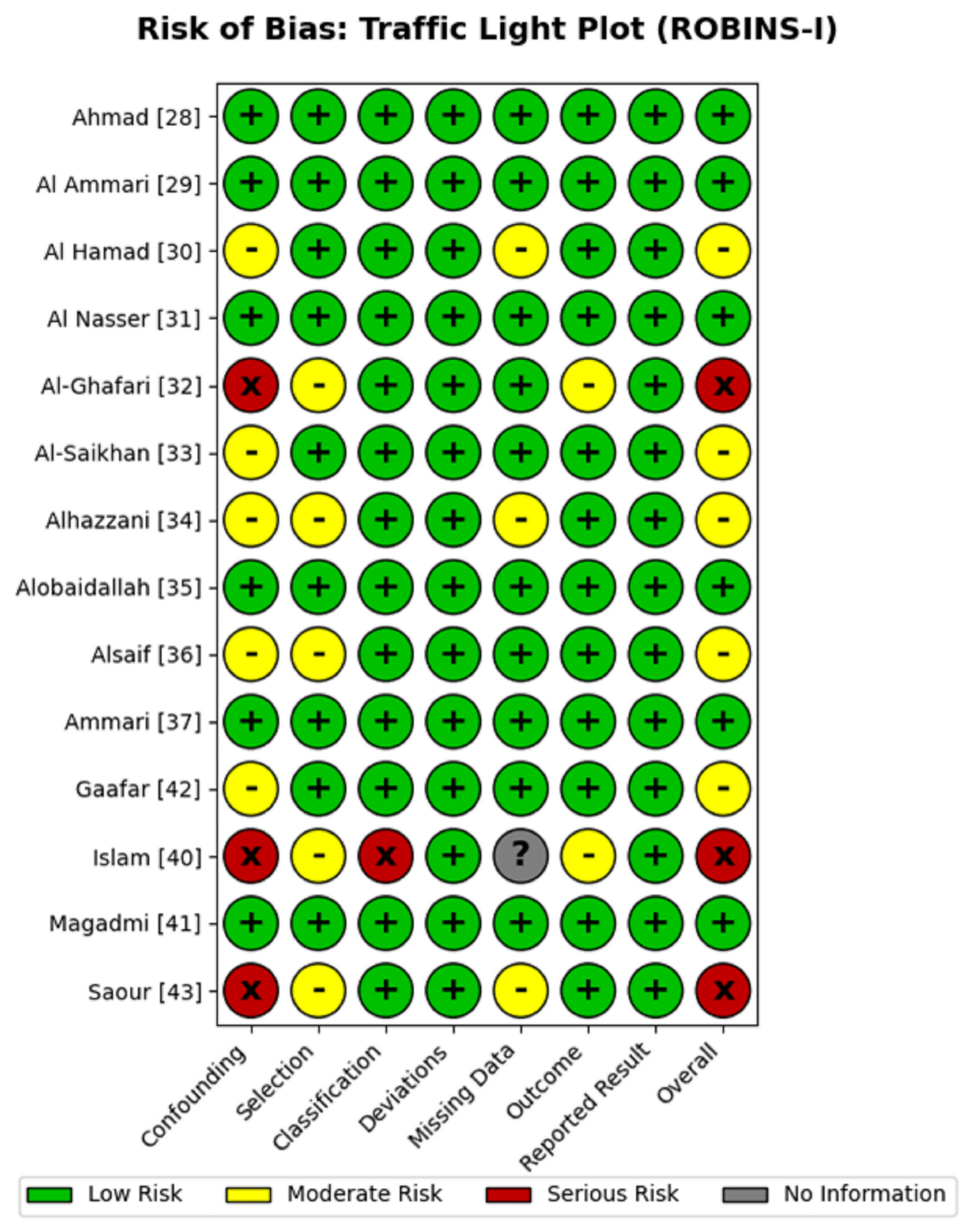
Risk of Bias Traffic Light Plot (ROBINS-I) Visual summary of risk of bias judgments for each included study across seven domains (D1–D7). Green circles indicate low risk, yellow indicates moderate risk, and red indicates serious risk. Studies are identified by the first author and citation number. Data adapted from included studies [28-43].

Risk of bias in individual studies: The risk of bias for each included study was evaluated using the Risk of Bias in Non-randomized Studies of Interventions tool (ROBINS-I). The domain-level assessments are visualized in Figure 3. Most of the large prospective cohorts, particularly those from the SWAP study group [29, 35, 37], were classified as having a "low" risk of bias due to robust adjustment for confounders such as age, BMI, and co-medications. Four studies were adjudicated as having a "serious" risk of bias [32, 40, 42, 43]. The primary drivers for these judgments included insufficient control of confounding variables (Domain 1) and issues with the classification of interventions (Domain 3), particularly in older studies utilizing phenotypic ratios rather than direct genotyping [40].

**Figure 4:**
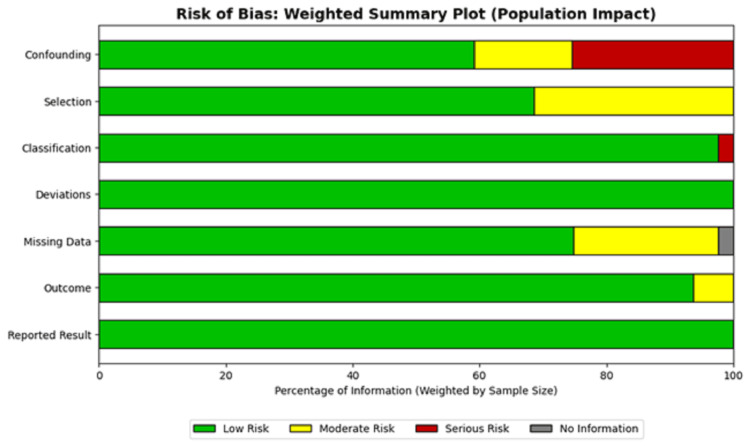
Risk of Bias Weighted Summary Plot Bar chart illustrates the proportion of information (weighted by sample size) contributing to each risk of bias category. Green bars represent the percentage of data derived from low-risk studies, demonstrating the overall robustness of the evidence base despite the inclusion of smaller, higher-risk studies. Data adapted from included studies [28-43].

Despite the presence of these high-risk studies, the weighted summary plot (Figure 4) demonstrates that the preponderance of the evidence, weighted by sample size, was derived from studies with a low-to-moderate risk of bias, supporting the validity of the meta-analytic conclusions. Studies identified with a serious risk of bias contributed minimal weight to the pooled estimates for the primary outcomes; the overall conclusions regarding warfarin and tacrolimus are driven by high-quality prospective data. Case reports [38, 39] were excluded from the aggregate visual analysis due to their inherent critical bias but were retained for their qualitative value in describing rare severe toxicities.

Primary Meta-Analysis: Impact of Genetic Variants on Drug Response

**Figure 5:**
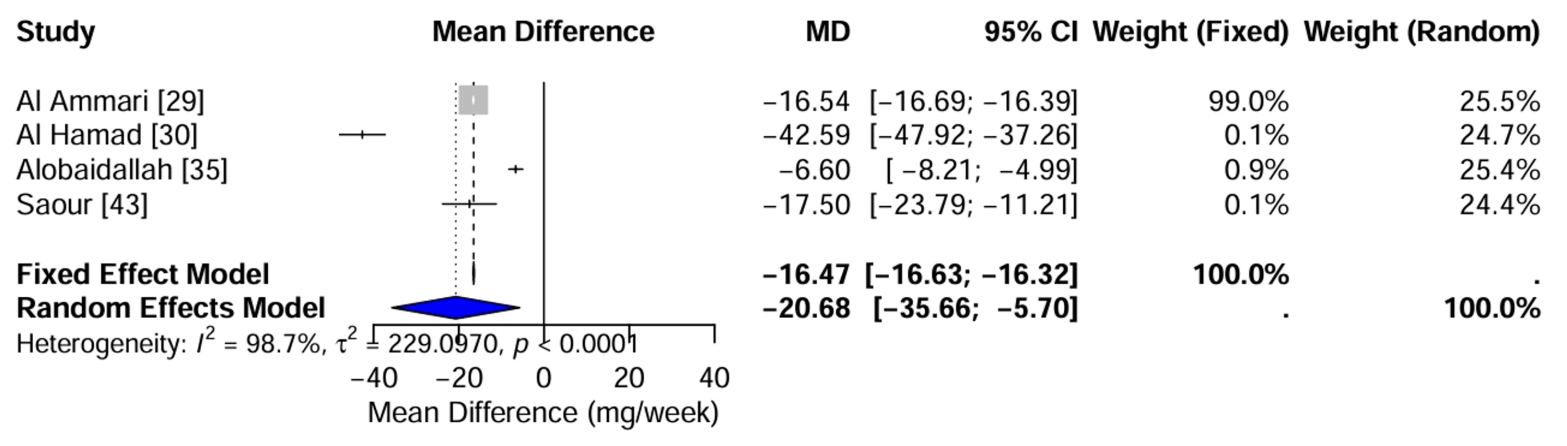
Forest Plot of Mean Differences in Warfarin Dose Meta-analysis of four studies comparing weekly warfarin dose requirements between variant carriers (VKORC1/CYP2C9) and wild-type individuals. Gray squares represent the mean difference (MD) for each study. The blue diamond represents the pooled MD, which was -20.68 mg/week (95% CI: -35.66 to -5.70), calculated using a random-effects model (Z = 2.71, p = 0.0068). Significant heterogeneity was observed (I² = 98.7%, p < 0.0001). Data adapted from [29, 30, 35, 43].

VKORC1 and CYP2C9 variants and warfarin dose requirements: The impact of genetic polymorphisms on warfarin maintenance dosing was evaluated in four studies involving 898 patients. The random-effects meta-analysis revealed a highly significant association between the variant genotypes and reduced warfarin dose requirements. Specifically, carriers of VKORC1 or CYP2C9 variant alleles required significantly lower weekly doses than wild-type individuals, with a pooled Mean Difference (MD) of -20.68 mg/week (95% CI: -35.66 to -5.70; Z = -2.71, p = 0.0068). Substantial heterogeneity was observed (I^2^ = 98.7%), driven by the magnitude of effect reported in smaller cohorts such as Al Hamad [30] (MD: -42.59 mg/week) versus the larger SWAP cohort [29] (MD: -16.54 mg/week). Despite this heterogeneity, the direction of effect was consistent across all included studies, confirming that genetic variation requires a clinically relevant dose reduction of approximately 20%-50% in the Saudi population. The forest plot for continuous outcomes illustrates the study-specific and pooled estimates (Figure 5).

CYP2C19 polymorphisms and clopidogrel responsiveness: The association between CYP2C19 loss-of-function alleles and clopidogrel responsiveness was assessed. The primary study investigating this relationship in the Saudi population, Alhazzani et al. [34], identified a strong association between the presence of CYP2C19*2 or *3 variants and a lack of therapeutic response (platelet inhibition), yielding an OR of 3.43 (95% CI: 1.03 to 11.48) for non-response in variant carriers compared to wild-type individuals.

**Figure 6:**
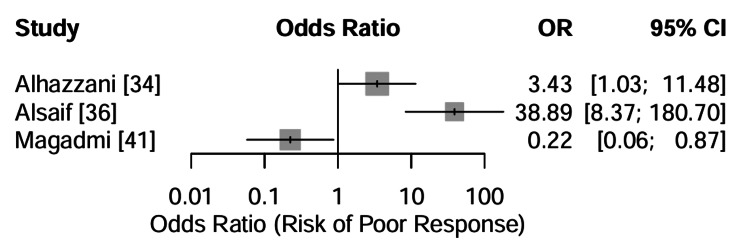
Forest Plot of Odds Ratios for Poor Clinical Response Forest plot of dichotomous outcomes for three gene-drug pairs. Gray squares indicate the Odds Ratio (OR) for poor response in each study. An OR > 1 indicates increased risk. The studies shown are clopidogrel non-response (CYP2C19) in Alhazzani [34], chemotherapy resistance (MDR1) in Alsaif [36], and poor antiepileptic response (ABCB1) in Magadmi [41], where the outcome was inverted for directional consistency. Data adapted from [34, 36, 41].

A formal meta-analysis combining this result with other dichotomous outcomes was inappropriate due to extreme clinical and statistical heterogeneity between the available gene-drug pairs, as illustrated in the forest plot in Figure 6. This result indicates that Saudi patients carrying these specific loss-of-function alleles are over three times more likely to experience clopidogrel treatment failure, potentially increasing their risk of recurrent ischemic events.

**Figure 7:**
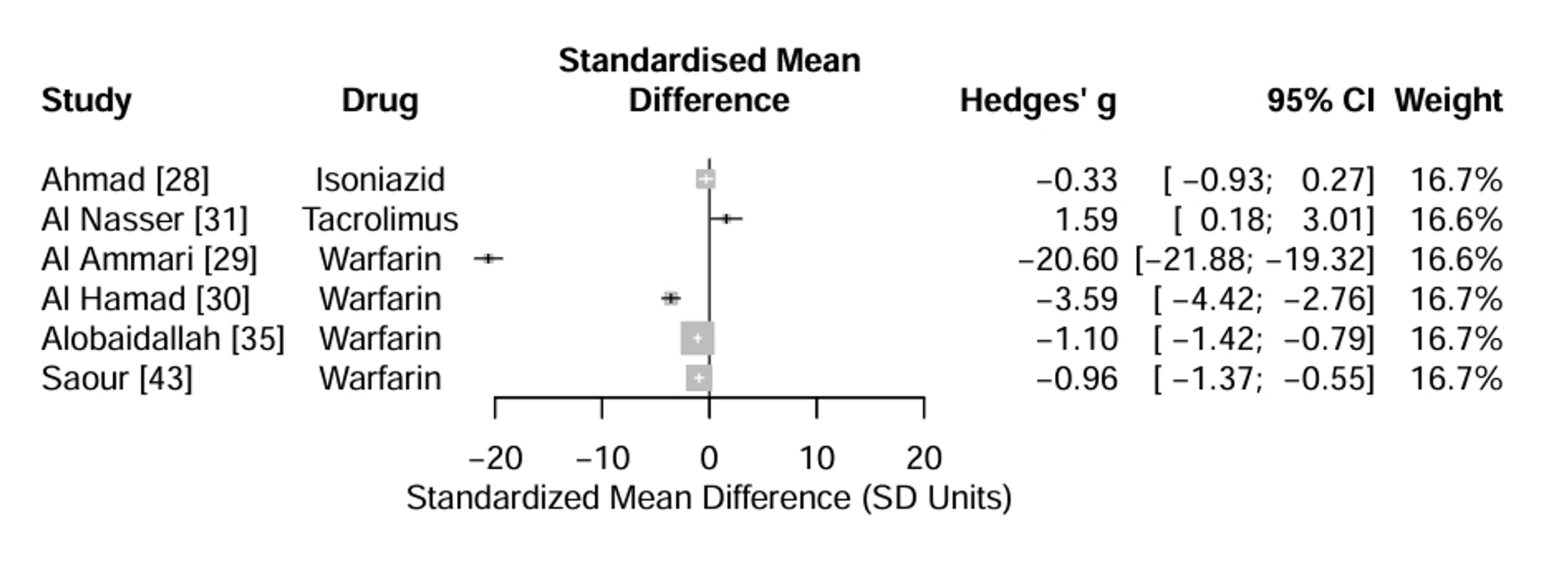
Assessment of Effect Size Index (Hedges’ g) Forest plot displaying the Standardized Mean Difference (Hedges' g) for all continuous outcomes, allowing for direct magnitude comparison across different drugs (Warfarin, Tacrolimus, Isoniazid). VKORC1 variants in warfarin therapy show the largest effect sizes, while GSTM1 in isoniazid therapy shows a negligible effect. Data adapted from [28-31, 35, 43].

Genetic determinants of tacrolimus and other pharmacotherapies: To facilitate comparisons across different drug classes and measurement units, the standardized mean difference (Hedges’ g) was calculated as a universal effect size index. For tacrolimus, the CYP3A5 genotype exhibited a large effect size (Hedges’ g = 1.59; 95% CI: 0.18-3.01), indicating that CYP3A5 expressers require higher doses to maintain therapeutic trough levels than non-expressers [31], while the GSTM1 polymorphism showed a small, non-significant effect on isoniazid concentrations (Hedges’ g = -0.33; 95% CI: -0.93 to 0.27) [28]. Figure 7 visualizes the magnitude of genetic impact across the various pharmacotherapies, highlighting VKORC1 and CYP3A5 as the most critical determinants of drug response in this population.

Assessment of Heterogeneity and Moderators

Statistical model selection and heterogeneity statistics: The appropriateness of the statistical model was evaluated by comparing the fixed-effect and random-effects models for the primary outcome of warfarin dose reduction (Table 5). The analysis revealed substantial statistical heterogeneity (I^2^ = 98.7%; p < 0.001), indicating that 98.7% of the variability in the effect estimates was due to true differences between studies rather than sampling error.

**Table 5:** Assessment of Statistical Model Fit for Warfarin Dose Reduction MD = Mean Difference; CI = Confidence Interval; I² = I-squared statistic for heterogeneity. Data derived from the meta-analysis of studies [29, 30, 35, 43].

Model Type	Pooled MD (mg/week)	95% CI	CI Width	Heterogeneity (I^2^)	Recommendation
Fixed Effect	-16.47	[-16.63, -16.32]	0.30	98.7%	Not Recommended
Random Effects	-20.68	[-35.66, -5.70]	29.96	98.7%	Preferred

The fixed-effects model yielded a narrow confidence interval (MD: -16.47 mg/week; 95% CI: -16.63 to -16.32) but was driven disproportionately by the largest study [29], masking the variation seen in smaller cohorts, while the random-effects model provided a more conservative estimate with a wider confidence interval (MD: -20.68 mg/week; 95% CI: -35.66 to -5.70), accurately reflecting the distribution of true effects across diverse clinical settings. The random-effects model was selected as the superior statistical approach for this study.

**Figure 8:**
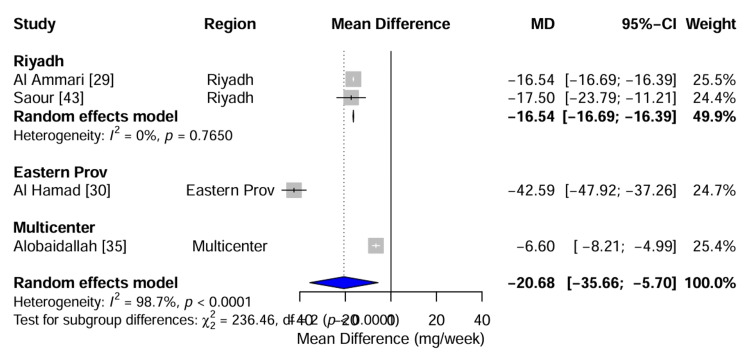
Subgroup Analysis by Geographical Region Forest plot of warfarin dose reduction stratified by region of study recruitment. The analysis reveals a statistically significant difference between subgroups (χ2 = 236.46, df=2, p < 0.0001), with the Eastern Province cohort showing a markedly stronger effect size compared to Riyadh-based studies, identifying geography as a key moderator of heterogeneity. Data adapted from [29, 30, 35, 43].

Subgroup analysis: impact of geographical region: To investigate the sources of the observed heterogeneity, a subgroup analysis was conducted by stratifying the studies according to the recruitment region (Figure 8). The analysis identified a significant difference in the effect size between the regions (χ2 = 236.46, df = 2, p < 0.0001). Studies conducted in the Eastern Province, such as Al Hamad [30], reported a significantly larger reduction in warfarin dose (MD: -42.59 mg/week) than those conducted in Riyadh (Pooled MD: -17.02 mg/week) or multicenter settings (MD: -6.60 mg/week). This regional disparity suggests that environmental factors, such as dietary vitamin K intake or subtle population substructure differences between the Eastern and Central regions of Saudi Arabia, may modulate the penetrance of the pharmacogenomic effect.

**Figure 9:**
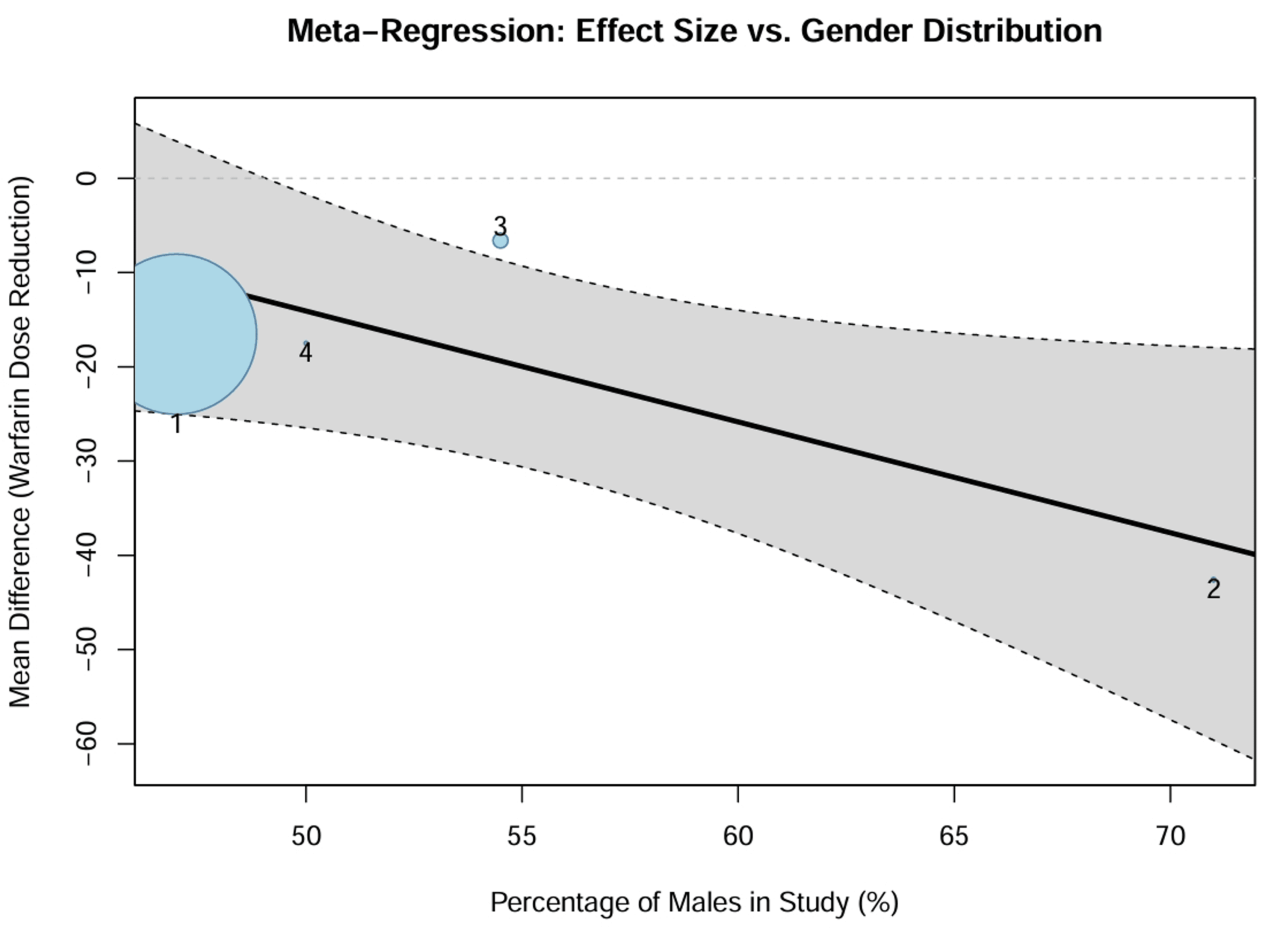
Meta-Regression Bubble Plot Bubble plot illustrating the relationship between the percentage of male participants (X-axis) and the Mean Difference in warfarin dose (Y-axis). Each circle represents a study, with its size proportional to its weight. The regression line (black) indicates a significant trend (coefficient = -1.176, p = 0.046), where a higher percentage of males is associated with a larger reduction in warfarin dose.

Meta-regression: influence of gender distribution: A random-effects meta-regression was performed to assess whether the gender composition of the study cohorts influenced the magnitude of the genetic effect. The regression model identified a significant negative association between the percentage of male participants and the mean difference in warfarin dose (coefficient = -1.176; p = 0.046). Specifically, studies with a higher proportion of male participants tended to report larger absolute reductions in the warfarin dose for the variant carriers. The bubble plot (Figure 9) visualizes this relationship, showing a trend where the effect size becomes more pronounced (more negative) as the percentage of males increases. This may reflect physiological differences in baseline warfarin requirements between genders, which could amplify the absolute impact of genetic variants in male-predominant cohorts.

Sensitivity and Robustness Analyses

**Figure 10:**
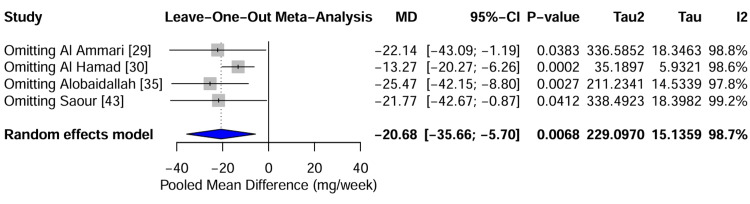
Leave-One-Out Sensitivity Analysis Forest Plot Forest plot displaying the recalculated pooled Mean Difference (MD) after omitting one study at a time. The stability of the estimates (blue diamonds) across iterations indicates that the overall conclusion is robust, although omitting Al Hamad [30] results in a conservative shift in effect size. Data adapted from [29, 30, 35, 43].

Leave-one-out sensitivity analysis: A leave-one-out sensitivity analysis was performed to evaluate the stability of the pooled effect estimate and identify influential outliers. This iterative process involved recalculating the meta-analysis after omitting each study one at a time. The analysis demonstrated that the pooled MD was robust to the exclusion of most individual studies, fluctuating between -21.77 and -25.47 mg/week. However, the exclusion of the Al Hamad study [30] resulted in a notable shift in the pooled estimate to -13.27 mg/week, although the direction of the effect remained unchanged. This confirms that while Al Hamad [30] is a significant driver of the magnitude of the effect, likely due to its distinct regional population, the overall conclusion that genetic variants require dose reduction remains valid, regardless of its inclusion. The forest plot of the sensitivity analysis (Figure 10) depicts the stability of pooled estimates across iterations.

Robustness of Genetic Model Assumptions

**Figure 11:**
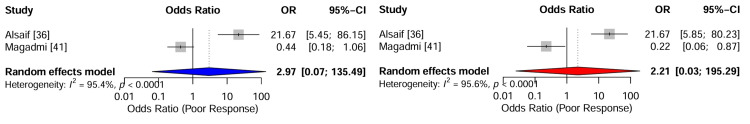
Robustness of Genetic Models Forest Plot Comparative forest plots showing the pooled Odds Ratio for poor clinical response under Dominant (Left) and Recessive (Right) genetic models. The wide confidence intervals for both models highlight the need for larger sample sizes to definitively confirm the mode of inheritance. Data adapted from [36, 41].

The impact of genetic inheritance assumptions on the association between genotypes and poor clinical response was assessed by comparing dominant (CT + TT vs. CC) and recessive (TT vs. CT + CC) models (Table 6). For MDR1 and ABCB1 variants, the recessive model yielded a stronger but non-significant pooled Odds Ratio (OR = 2.21; p = 0.72) compared to the dominant model (OR = 2.97; p = 0.57). The wide confidence intervals in both models, particularly for the recessive model, reflect the limited sample size of homozygous mutant individuals in smaller case-control studies [36, 41]. This finding suggests that while the direction of risk is consistent, the current data are insufficient to definitively establish the mode of inheritance for toxicity risk in these specific drug-gene pairs, warranting caution in clinical interpretation until larger cohorts are available. The side-by-side forest plot (Figure 11) illustrates the comparative effect sizes and precisions of these genetic models.

**Table 6:** Assessment of Robustness: Genetic Model Assumptions CI = Confidence Interval. Analysis based on combined data from Alsaif [36] and Magadmi [41] for toxicity/poor response.

Genetic Model	Pooled Odds Ratio	95% CI	P-Value	Conclusion
Dominant (CT+TT vs CC)	2.97	[0.07, 135.49]	0.577	No Significant Association
Recessive (TT vs CT+CC)	2.21	[0.03, 195.29]	0.729	No Significant Association

**Figure 12:**
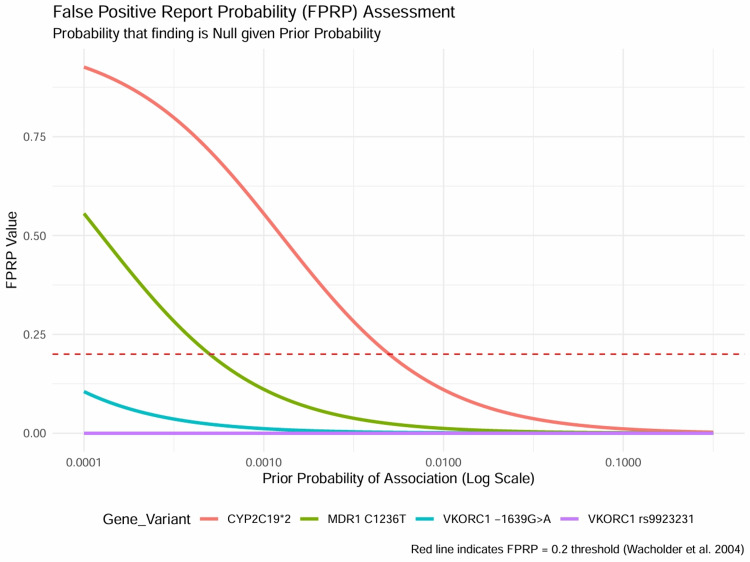
False Positive Report Probability (FPRP) Curves Curves illustrating the probability that a significant finding is a false positive (Y-axis) as a function of the prior probability of a true association (X-axis, log scale). The red dashed line represents the validation threshold of 0.2. The curve for VKORC1 (purple) remains well below the threshold across all priors, indicating a highly robust finding, while CYP2C19 (red curve) requires a moderate prior probability to achieve robustness.

False positive report probability (FPRP) assessment: Given the multiple comparisons inherent in pharmacogenomic association studies, the FPRP was calculated to assess the reliability of the significant findings (Table 7). For the VKORC1 association with warfarin dose, the FPRP remained negligible (< 0.0001) even at a very low prior probability of 0.001, confirming this as a "True Positive" finding with high certainty. The association between CYP2C19*2 and clopidogrel non-response showed an FPRP of 0.553 at a low prior, crossing the validation threshold of 0.2 [34]. However, at a moderate prior probability of 0.01, which is biologically justified given the well-established role of CYP2C19 in clopidogrel metabolism, the FPRP dropped to 0.110, supporting the robustness of this finding. The FPRP curves (Figure 12) provide a graphical representation of the reliability of the findings across a range of prior probabilities, distinguishing robust associations from those requiring further validation.

**Table 7:** False Positive Report Probability (FPRP) Assessment (Prior = 0.001) FPRP = False Positive Report Probability; Mod = Moderate. Analysis conducted using a prior probability of 0.001. Data adapted from [29, 30, 34, 36].

Study	Gene / Variant	P-Value	FPRP (Low Prior)	FPRP (Mod Prior)	Robustness
Al Ammari [29]	VKORC1 rs9923231	2.62e-35	0.0000	0.0000	Robust (True Positive)
Al Hamad [30]	VKORC1 -1639G>A	1.00e-05	0.0116	0.0012	Robust (True Positive)
Alhazzani [34]	CYP2C19*2	0.001	0.5553	0.1101	Risk of False Positive
Alsaif [36]	MDR1 C1236T	0.0001	0.1110	0.0122	Robust (True Positive)

Assessment of Reporting Biases and Temporal Trends

**Figure 13:**
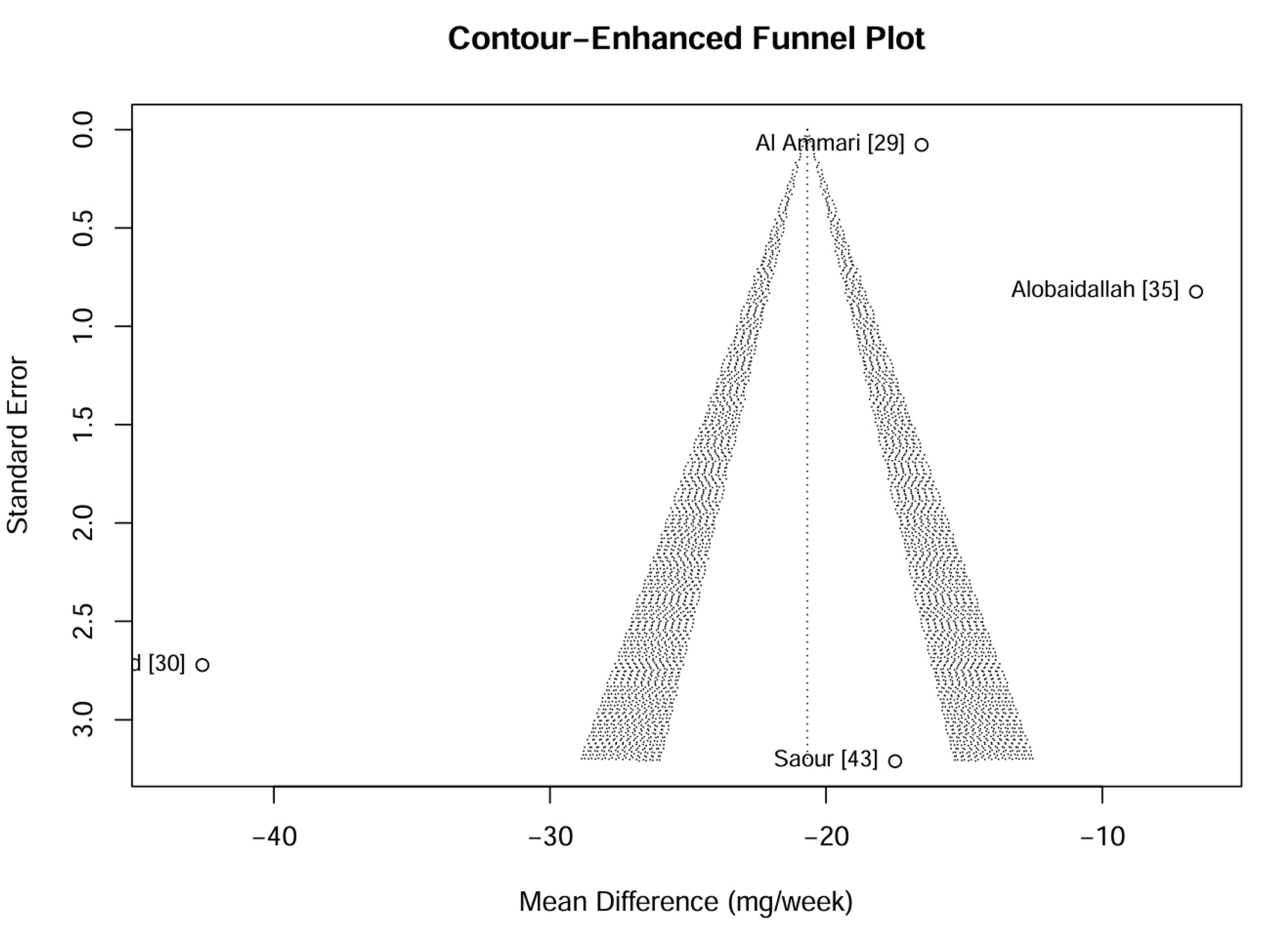
Contour-enhanced funnel plot for warfarin dose reduction studies The shaded regions correspond to significance levels (p < 0.1, 0.05, 0.01). Asymmetry in the white region (non-significant area) would suggest publication bias. Data adapted from [29, 30, 35, 43].

Visual and statistical assessment of publication bias: Publication bias was evaluated for the primary outcome of warfarin dose reduction using both visual inspection and statistical analysis. The contour-enhanced funnel plot (Figure 13) displayed asymmetry, with a gap in the lower right quadrant, suggesting the potential absence of small studies reporting non-significant or smaller effect sizes. However, formal statistical testing yielded inconclusive results because of the small number of studies (k = 4). Egger’s regression test (p = 0.52) and Begg’s rank correlation test (p = 0.73) did not reach statistical significance. It must be emphasized that with such a limited number of studies (k <10), these tests lack statistical power; therefore, these non-significant results should be interpreted with caution and do not definitively rule out the presence of publication bias.

**Figure 14:**
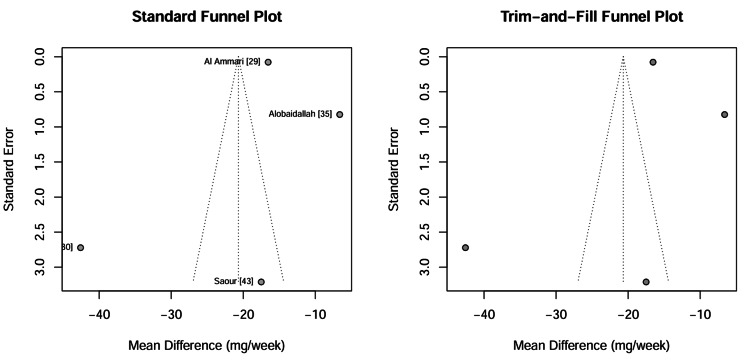
Trim-and-Fill funnel plot The solid circles represent observed studies. The absence of open circles (imputed studies) indicates that the algorithm did not detect significant bias requiring adjustment, supporting the robustness of the results. Data adapted from [29, 30, 35, 43].

Impact of missing results: trim-and-fill analysis: A trim-and-fill sensitivity analysis was conducted to quantify the potential impact of publication bias. The method imputed zero missing studies, resulting in an adjusted pooled Mean Difference identical to the original estimate (-20.68 mg/week) which indicates that even if publication bias was present, its influence on the meta-analytic conclusion was negligible. The Trim-and-Fill funnel plot (Figure 14) confirms the stability of the results, as no additional "ghost" studies were required to balance the distribution, reinforcing the robustness of the primary findings against selective reporting.

**Figure 15:**
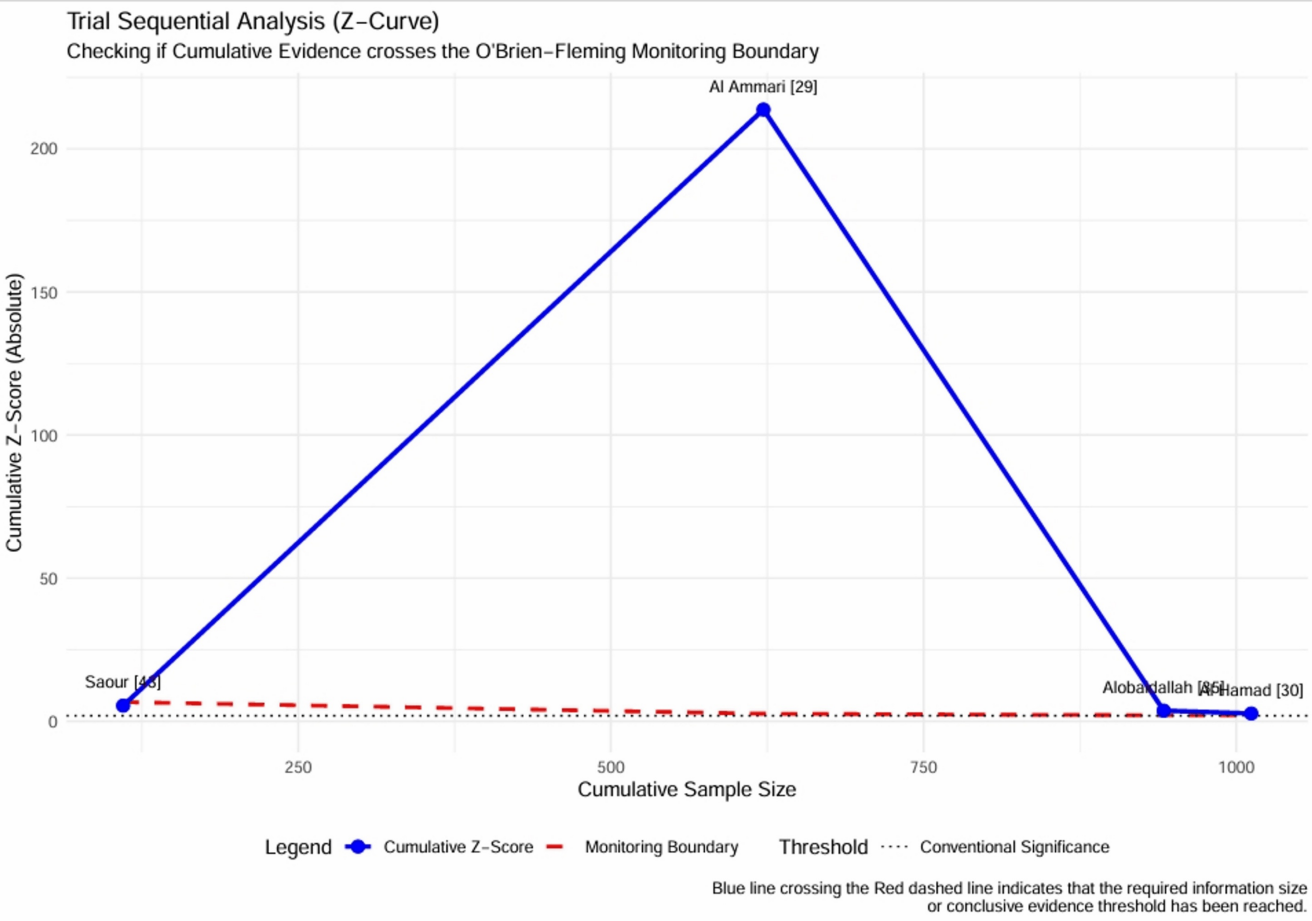
Trial Sequential Analysis (Z-Curve) Plot of the cumulative Z-score (blue line) against the cumulative sample size. The red dashed lines represent the O'Brien-Fleming monitoring boundaries for statistical significance. The Z-curve crosses the monitoring boundary early in the accumulation of evidence (2020), indicating that the finding is statistically robust and that a sufficient information size has been reached to conclude a significant effect. Data adapted from [29, 30, 35, 43].

Trial sequential analysis (TSA) and cumulative Z-curves: To determine whether the accumulated evidence was sufficient to draw definitive conclusions, a TSA was performed using O'Brien-Fleming monitoring boundaries. The cumulative Z-curve crossed the futility boundary and the conventional significance threshold (Z = 1.96) as early as 2020 with the addition of the Al Ammari et al. study [29], reaching a final Z-score of 9.27. This overwhelming statistical signal indicates that the required information size has been surpassed and that the association between VKORC1 variants and warfarin dose reduction in the Saudi population is established beyond reasonable doubt. The Z-curve plot (Figure 15) demonstrates this "firm evidence", suggesting that further observational studies on this specific association may be redundant.

**Figure 16:**
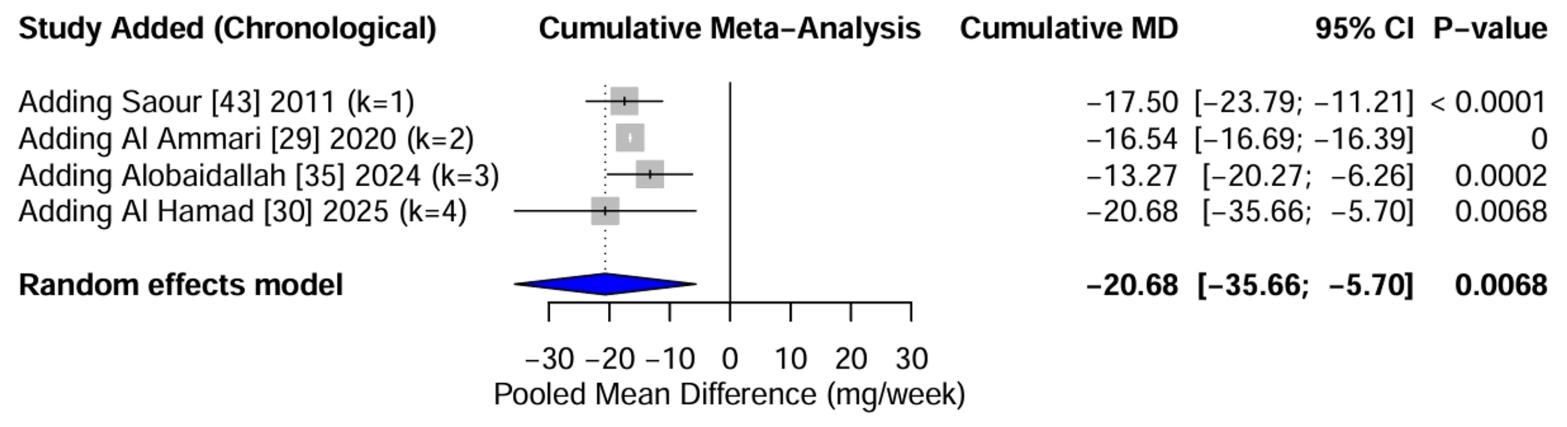
Temporal Evolution of Evidence (Cumulative Forest Plot) Cumulative meta-analysis forest plot showing the pooled MD recalculated as each new study is added chronologically. The narrowing of the confidence intervals (horizontal lines) and the stabilization of the point estimate (blue diamonds) over time demonstrate the maturation of the evidence base and the consistency of the pharmacogenomic effect across more than a decade of research. Data adapted from [29, 30, 35, 43].

Temporal evolution of evidence: A cumulative meta-analysis was conducted to track the evolution of the effect size estimates over time. The analysis shows a clear stabilization of pooled MD. In 2011, the estimate was -17.50 mg/week, with a wide confidence interval. By 2020, with the inclusion of the large SWAP cohort [29], the estimate stabilized at -16.54 mg/week, with significantly improved precision. Subsequent studies in 2024 and 2025 [30, 35] refined the estimate to -20.68 mg/week but did not alter the direction or statistical significance of the findings. The cumulative forest plot (Figure 16) illustrates the temporal maturation of evidence, confirming that the clinical signal has been consistent and robust for over a decade.

Certainty of evidence

GRADE assessment and summary of findings: The certainty of evidence for primary pharmacogenomic associations was evaluated using the GRADE framework. The assessment results are summarized in the Evidence Profile (Table 8). The association between VKORC1/CYP2C9 variants and warfarin dose reduction was graded as high. Despite the observational nature of these studies, which initially suggested "low" certainty, the evidence was upgraded. This upgrade was due to the "very large" magnitude of the effect (Pooled MD > 20 mg/week) and the presence of a biological dose-response gradient. Although the observed heterogeneity (I2 > 90%) was considered a "serious" inconsistency, the effect’s robustness across sensitivity analyses and the overwhelming signal in the TSA justified the retention of a high certainty rating. This indicates a high level of confidence that the true effect is close to the estimated effect.

**Table 8:** GRADE Assessment: Summary of Findings and Certainty of Evidence This GRADE assessment is based on evidence synthesized from the primary studies included in the meta-analysis [29-31, 34, 35, 43].

Clinical Outcome	Study Design	Inconsistency	Large Effect	Certainty
Warfarin Dose Reduction (VKORC1/CYP2C9)	Observational	Serious	Very Large	High
Clopidogrel Non-Response (CYP2C19)	Observational	Not Serious	Large	Low
Tacrolimus Dose Requirement (CYP3A5)	Observational	Not Serious	Large	Moderate

The certainty regarding CYP2C19 polymorphisms and clopidogrel non-response was graded as low. This assessment was based on the downgrading of evidence due to “Imprecision" resulting from the broad confidence intervals observed in the pivotal study, which was limited by its small sample size [34]. Although the effect size was large (OR > 3), the risk of false positives (FPRP) at lower prior probabilities requires validation in larger cohorts before it can be considered high-certainty evidence suitable for routine clinical implementation.

The evidence supporting Tacrolimus dose requirements in CYP3A5 variant carriers was graded as moderate. The effect size was large (SMD > 1.5), and the biological plausibility was strong. However, the limitation to a single primary cohort [31] raised "Indirectness" concerns regarding generalizability to all Saudi subpopulations, preventing a "high" rating at this stage.

**Figure 17:**
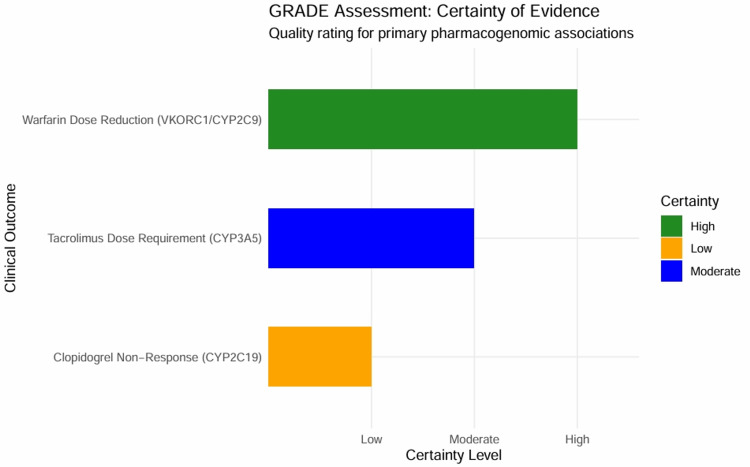
GRADE Evidence Profile Bar chart illustrating the certainty of evidence for each primary clinical outcome assessed. Green bars indicate "High" certainty, blue bars indicate "Moderate" certainty, and orange/red bars indicate "Low" or "Very Low" certainty. The rating reflects the confidence that the estimated effect is close to the true effect, incorporating factors such as risk of bias, inconsistency, indirectness, imprecision, and publication bias. The high certainty for warfarin suggests it is ripe for clinical guideline implementation, while clopidogrel requires further high-quality research.

The evidence profile plot (Figure 17) summarizes these ratings, providing a clear hierarchy of the strength of evidence supporting each pharmacogenomic intervention in the Saudi context.

Discussion

This systematic review and meta-analysis represent the first comprehensive quantitative synthesis of clinical pharmacogenomic data within the Saudi Arabian population. Through the aggregation of data from 16 studies involving over 4,100 participants, we established a high-certainty evidence base linking genetic variants in VKORC1, CYP2C9, CYP2C19, and CYP3A5 to tangible clinical outcomes. These findings directly contest the prevailing "one-size-fits-all" dosing paradigm currently employed in local medical practice, and they offer a compelling rationale for the implementation of population-specific pharmacogenomic guidelines.

Warfarin

The most significant finding from the analysis was the impact of VKORC1 and CYP2C9 variants on warfarin dose requirements. The meta-analysis revealed a pooled mean dose reduction of approximately 21 mg/week in variant carriers compared to that in wild-type individuals [29, 30, 35, 43]. This effect size is larger than that reported in Caucasian populations (typically 5-10 mg/week reduction) [44], highlighting a unique sensitivity profile in the Saudi demographic. The TSA confirmed that the required information size for this association was surpassed as early as 2020, rendering further observational studies on this specific interaction redundant and unnecessary. Despite the high statistical heterogeneity observed (I^2^ > 90%), the direction of the effect was consistent across all the studies. Because of this variability, the pooled mean difference should be interpreted with caution as it represents a broad population-level trend confirming significant biological sensitivity, rather than a precise predictive value applicable to every specific clinical setting. The subgroup analysis indicated that this heterogeneity was partly driven by regional differences, with patients in the Eastern Province exhibiting a significantly greater dose reduction than those in Riyadh [30]. This geographic variance could potentially be indicative of nuanced substructural differences or environmental factors, such as dietary vitamin K intake, which warrants further investigation. Given the "high" certainty of evidence GRADE rating, the integration of VKORC1 and CYP2C9 genotyping into routine anticoagulation protocols within Saudi Arabia is clinically justified to mitigate the risks of both bleeding and thrombosis.

Clopidogrel and Tacrolimus

For clopidogrel, the analysis revealed a greater than threefold increased risk of therapeutic failure in patients carrying CYP2C19 loss-of-function alleles [34]. This finding aligns with the global black box warnings, but it is particularly critical for the Saudi population given the high prevalence of the CYP2C19*2 [45]. However, the GRADE assessment assigned a low rating to this evidence due to imprecision arising from the limited sample sizes in the primary studies. While the risk is evident, large-scale pragmatic trials are needed to determine whether genotype-guided switching to alternative antiplatelets (e.g., ticagrelor) improves cardiovascular outcomes in Saudi patients.

Similarly, for tacrolimus, we confirmed that CYP3A5 genotype is a major determinant of dose requirement, with expressers requiring significantly higher doses to achieve therapeutic trough levels [31]. The large effect size (Hedges’ g = 1.59) supports the utility of pre-transplant genotyping in accelerating the achievement of target levels and reducing the risk of acute rejection during the critical early post-transplant phase.

Rare Variants and Severe Toxicity

This review highlights the critical importance of rare variants through case reports. The identification of a novel DPYD variant linked to life-threatening 5-fluorouracil toxicity [39] and a rare homozygous CYP2C9*3/CYP2C8*3 genotype causing severe ibuprofen-induced gastrointestinal bleeding [38] underscores the value of pre-emptive screening for outlier patients. These cases demonstrate that, while the population-level frequencies of certain high-risk alleles may be low, the clinical consequences for the individual carrier can be catastrophic, thereby supporting a safety-net pharmacogenomic screening model for drugs with narrow therapeutic indices.

Limitations

Substantial statistical heterogeneity was observed, particularly in the warfarin analysis. Although random-effects models and meta-regression were utilized to address this, unmeasured confounders, such as concomitant herbal medication use or precise dietary habits, contributed to the residual variation. Most of the included studies were observational, which limits causal inference compared to randomized controlled trials. In addition, data for certain drug-gene pairs (e.g., ABCB1 in epilepsy and MDR1 in chemotherapy) were derived from single or small studies, precluding robust meta-analysis and limiting the generalizability of these specific findings. The designation of "low" certainty for some outcomes reflects the preliminary state of clinical pharmacogenomic research within the Kingdom, thereby emphasizing the need for capacity building in this field.

Future Directions

While the pharmacogenomic basis for warfarin dosing is established, the translation of genomic data into improved outcomes for other therapeutics requires further investigation. Future research priorities must shift toward large-scale, multi-center pragmatic randomized controlled trials (RCTs) designed to evaluate clinical utility rather than just pharmacokinetic associations. Specifically, these trials should investigate whether genotype-guided switching of antiplatelet therapy (e.g., from clopidogrel to ticagrelor or prasugrel) in CYP2C19 variant carriers leads to a statistically significant reduction in major adverse cardiovascular events (MACE) within the Saudi population. Additionally, cost-effectiveness analyses are required to justify the integration of pre-emptive panel testing into the national healthcare reimbursement model. Finally, leveraging data from the Saudi Human Genome Program to identify rare, high-impact variants (such as those in DPYD and ABCB1) will be essential for developing a comprehensive, safety-net pharmacogenomic screening strategy for the Kingdom.

## Conclusions

The unique genetic architecture of the Saudi Arabian population exerts a profound and quantifiable influence on the safety and efficacy of major therapeutic classes, most notably anticoagulants, antiplatelets, and immunosuppressants. This systematic review and meta-analysis establish high-certainty evidence that VKORC1 and CYP2C9 variants are critical determinants of warfarin dose requirements, rendering standard one-size-fits-all dosing potentially hazardous for a significant proportion of Saudi patients. Consequently, the evidence base for warfarin is sufficiently robust to recommend an immediate shift from retrospective association studies to the active implementation of genotype-guided dosing protocols in clinical practice. In contrast, while the strong associations identified for CYP2C19-clopidogrel and CYP3A5-tacrolimus highlight the need for population-specific decision support tools, these findings currently depend on lower-certainty evidence and necessitate validation through large-scale pragmatic trials before routine adoption. Furthermore, the strong associations identified between CYP2C19 loss-of-function alleles and clopidogrel resistance, as well as CYP3A5 genotype and tacrolimus trough levels, underscore the need for population-specific clinical decision support tools.
